# Screening Novel Furoxan Derivatives as Potential Inhibitors Targeting Thioredoxin Glutathione Reductase of *Fasciola gigantica*

**DOI:** 10.3390/ph18111603

**Published:** 2025-10-23

**Authors:** Yanhui Han, Yuting He, Qingqing Guo, Gongming Li, Huan Chen, Wenjiao Zhao, Yan Zhou, Zhiqiang Fu, Oyetunde T. Oyeyemi, Huili Zhu, Qiangqiang Wang, Dequn Sun, Yang Hong

**Affiliations:** 1 College of Animal Science and Technology, Henan Institute of Science and Technology, Xinxiang 453003, China; hanyanhui19860916@163.com (Y.H.); 888julia@163.com (H.Z.); vet_wang@126.com (Q.W.); 2 National Reference Laboratory for Animal Schistosomiasis, Key Laboratory of Animal Parasitology of Ministry of Agriculture and Rural Affairs, Shanghai Veterinary Research Institute, Chinese Academy of Agricultural Sciences, Shanghai 200241, China; 15382439442@163.com (Y.H.); qq18737197072@163.com (Q.G.); fuzhiqiang@shvri.ac.cn (Z.F.); 3 Marine College, Shandong University, Weihai 264209, China; xhlmwysf@sina.com (G.L.); 3181745@163.com (H.C.); wenjiao19940225@163.com (W.Z.); 4 National Institute of Parasitic Diseases, Chinese Center for Diseases Control and Prevention (Chinese Center for Tropical Diseases Research), National Health Commission of the People’s Republic of China (NHC) Key Laboratory of Parasite and Vector Biology, World Health Organization (WHO) Collaborating Center for Tropical Diseases, National Center for International Research on Tropical Diseases, Shanghai 200025, China; zhouyan@nipd.chinacdc.cn; 5 Tropical Medicine & Diagnostics Development (TMDD) Group, Department of Biosciences and Biotechnology, University of Medical Sciences, Ondo City 351001, Nigeria; ooyeyemi@unimed.edu.ng; 6 College of Life Sciences and Agri-Forestry, Southwest University of Science and Technology, Mianyang 621010, China; 7 Hainan Tropical Diseases Research Center, Haikou 571199, China

**Keywords:** *Fasciola gigantica*, thioredoxin glutathione reductase, enzymatic activity, furoxan

## Abstract

**Background:** Fascioliasis, caused by *Fasciola* species, is a significant public health concern affecting over 250 million people globally and causing annual economic losses exceeding USD 6 billion. The sole FDA-approved treatment, triclabendazole (TCZ), faces increasing resistance due to extensive use, highlighting the urgent need for alternative therapeutic targets. A promising candidate is thioredoxin glutathione reductase (TGR), a multifunctional enzyme unique to platyhelminths, essential for redox balance and parasite survival. **Methods:** This study investigated the antioxidant and enzymatic activities of recombinant *Fasciola gigantica* TGR (FgTGR), its localization within the parasite, and its inhibition by furoxan derivatives. FgTGRsec (FgTGR containing selenocysteine) was expressed and purified, and its enzymatic activities, including thioredoxin reductase (TrxR), glutathione reductase (GR), and glutaredoxin (Grx), were characterized. **Results:** Immunolocalization studies revealed FgTGR’s presence in critical tissues, underscoring its functional significance. Antioxidant assays demonstrated the protein’s role in protecting against oxidative damage. Inhibition assays with furoxan derivatives identified potential inhibitors targeting TGR activity. Sequence and phylogenetic analyses showed FgTGR’s evolutionary conservation among trematodes, confirming its potential as a drug target. **Conclusions:** The study’s findings establish FgTGR as a critical enzyme for parasite survival and a promising target for developing novel therapeutics. These results pave the way for the further screening and optimization of TGR inhibitors, offering a strategic approach to overcoming TCZ resistance and improving fascioliasis control.

## 1. Introduction

*Fasciola* infections contribute significantly to human disability and mortality in numerous developing countries, remaining one of the critical medical challenges of the 21st century [1,2,3]. The parasite infects about 250 million people worldwide and many domestic animals, with estimated economic losses of more than USD 6 billion every year [4]. Triclabendazole (TCZ) is the only drug of choice treatment for fascioliasis approved by the U.S. Food and Drug Administration (FDA) as it effectively targets both early immature and adult *Fasciola* worms [5]. To date, no new drug has been clinically introduced since the development of TCZ over 40 years ago. However, resistance to TCZ has been reported in sheep and cattle due to its widespread use [6,7]. This highlights the urgent need for the development of novel and effective drugs to ensure better control of fascioliasis in the future.

There are two redox systems in mammals. One is the glutathione (GSH) system containing glutaredoxin (Grx) and glutathione reductase (GR). The other is the thioredoxin (Trx) system containing Trx and thioredoxin reductase (TrxR). In both systems, the electrons are transferred from NADPH by using the enzymes GR and TrxR [8]. Thioredoxin glutathione reductase (TGR), a unique multifunctional enzyme, was recently discovered and replaced GR and TrxR enzymes in platyhelminths such as *Fasciola gigantica* (*F. gigantica*) [8,9,10], Schistosoma [11,12,13,14], and *Orientobilharzia turkestanicum* (*O. turkestanicum*) [15]. The TGR plays a crucial role in maintaining cellular redox balance, which is essential for the survival of *Fasciola* in the mammalian host. In *F. gigantica*, the sequence and domain structure of TGR are similar to those of other parasites, with typical CPYC and GCUG active sites. “U” is selenocysteine (Sec), and is able to confer unique properties to selenoprotein because of its high reactivity [16].

In recent years, TGR has gained recognition as a promising drug target for treating parasitic infections. In *Schistosoma mansoni* (*S. mansoni*), Kuntz et al. [12] demonstrated that SmTGR is critical for parasite survival, with parasites dying within four days in vitro following TGR silencing. In the same study, auranofin (AF) was proven to be an effective TGR inhibitor, successfully curing some partially infected mice. Additionally, potassium antimonyl tartrate (PAT) and oltipraz (OPZ) were previously utilized as anti-schistosomal drugs. The findings revealed that the compounds effectively inhibited TGR activity, confirming TGR as a critical target during treatment with these compounds. This study established SmTGR as the first validated key drug target for anti-schistosomiasis therapy [12]. In *Schistosoma japonicum* (*S. japonicum*), TGR could induce a partially protective effect against schistosome infection in mice. It also indicates that TGR is an effective candidate protein or a new drug target [13]. RNA interference studies have demonstrated that TGR plays a vital role in schistosome development [12,17]. In *Opisthorchis viverrini* (*O. viverrini*), TGR was successfully cloned, its enzymatic activities characterized, and its inhibition by AF, a well-known inhibitor, was confirmed. Additionally, AF exhibited toxic effects on both juvenile and adult worms in vitro. These findings suggest that OvTGR is essential for *O. viverrini* survival and represents a promising drug target for developing novel therapies against opisthorchiasis [18]. In schistosomes, AF also killed larval worms in vitro and could partially cure the infection [11,12,19]. Several other inhibitors were also identified for TGR besides AF [20,21,22,23,24,25]. These results validate TGR as a key drug target, and is an essential method to control the resistance of drugs by screening effective inhibitors of TGR.

In this work, antioxidant and enzymatic activities of FgTGRsec were analyzed, and the localization of FgTGR was detected by immunolocalization analysis. Several furoxan derivatives were tested for their inhibitory activity on TrxR. These results could provide a basis for screening FgTGR inhibitors and developing novel potential drug against fascioliasis in the future.

## 2. Results

### 2.1. Bioinformatics Analysis of FgTGR

Bioinformatics analysis revealed that there were two putative domains, N-terminal Grx and C-terminal TrxR, in the two recombinant proteins, FgTGR and FgTGRsec. In the N-terminal extension of the sequence, recombinant protein contained a glutaredoxin (Grx) domain including an active site CPYC. There was a pyridine nucleotide-disulfide active site (CVNVGC) after the sequence of Grx. Furthermore, the TrxR domain contained an NADH-binding site, FAD-binding domains, and a Sec-containing-GCUG- redox center in the C-terminus. The sequences analysis also demonstrated that the proteins did not possess a signal peptide.

**Figure 1:**
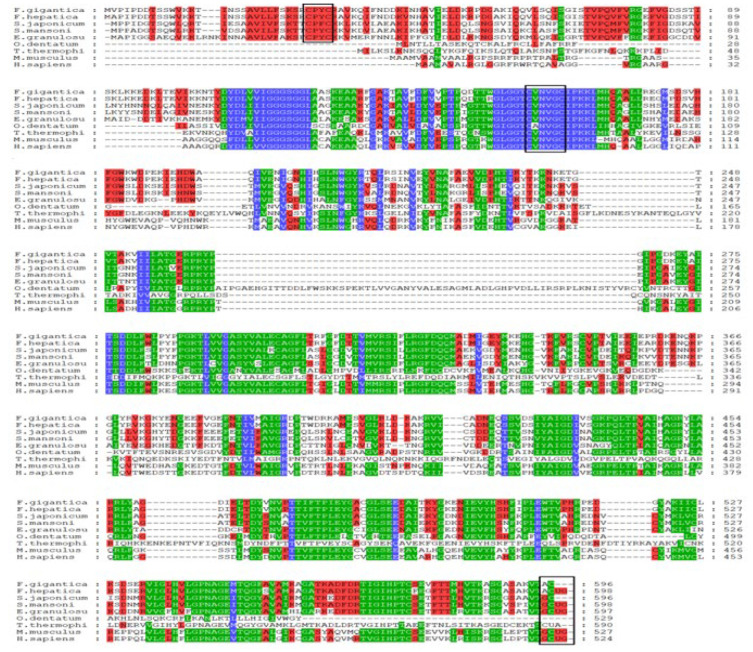
Alignment of the TGR amino acid sequence from *F. gigantica* with other TGRs and TR from some parasites: *F. hepatica* CAM96615.1, *S. mansoni* AAK85233.1, *S. japonicum* ACH86016.1, *E. granulosus* AAN63052.1, *T. thermophila* EAR83786.3, *O. dentatum* KHJ98088.1, *M. musculus* AAL90457.1, and *H. sapiens* AAD19597.1. The three conserved motifs are indicated by the black boxes. The different colors backgrounds indicated conserved and quantify mode colors.

**Figure 2:**
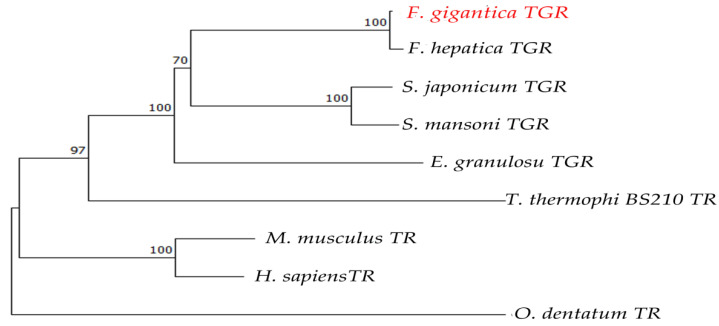
Phylogenetic tree analysis of homologs of TGRs and TR (the accession numbers of TGRs and TR are cited in Figure 1). The red color of *F. gigantica* TGR was used to highlight the target gene.

A comparative analysis of the FgTGR amino acid sequence with those of closely related parasitic species such as *Fasciola hepatica* (*F. hepatica*), *Echinococcus granulosus* (*E. granulosus*), *Schistosoma japonicum* (*S. japonicum*), *Schistosoma mansoni* (*S. mansoni*), *Tetrahymena thermohila* SB210 (*T. thermophila*), and *Oesophagostomum dentatum* (*O. dentatum*), along with two host species, *Homo sapiens* (*H. sapiens*) and *Mus musculus* (*M. musculus*), revealed that the TGR of *F. hepatica* exhibited the highest sequence identity (97.99%). FgTGR shared a different identity with others parasites such as *S. japonicum* (62.65%), *S. mansoni* (62.75%), and *E. granulosus* (58.36%). The conserved motifs “CPYC”, “GCUG”, and “CVNVGC” are marked with black boxes in Figure 1. In Figure 1, there are no CPYC amino acids sequences in *T. thermophila* and *O. dentatum*. The phylogenetic analyses of FgTGR are provided in Figure 2, showing that FgTGR is not closely related to nematodes and protozoans.

### 2.2. Supercoiled DNA Protection Activity of FgTGR

**Figure 3:**
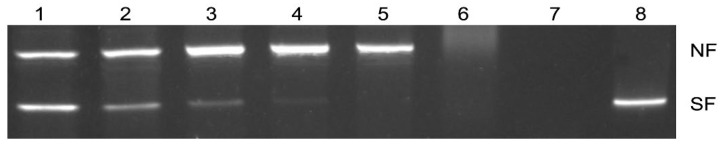
Protection of super-coiled DNA cleavage by FgTGR in an MCO system. Lanes (1–5) pUC19 plasmid + FeCl_3_ + DTT + FgTGR (12.5, 25, 50, 100, 200 μg/mL, respectively); (6) pUC19 plasmid + FeCl_3_ + DTT + 200 μg/mL BSA; (7) pUC19 plasmid + FeCl_3_ + DTT; (8) pUC19 plasmid. NF, nicked form of pUC19 plasmid; SF, super-coiled form of pUC19 plasmid.

The DNA protection activity of rFgTGR was evaluated using an MCO assay. In this assay, hydroxyl radicals induced nicking of the supercoiled pUC19 plasmid DNA, and the resulting DNA integrity was assessed by gel electrophoresis. The results demonstrated that rFgTGR effectively prevented plasmid DNA nicking, whereas no protective effect was observed in samples lacking FgTGR. In contrast, bovine serum albumin (BSA) used as a control protein did not prevent DNA damage. The protective effect of rFgTGR increased in a concentration-dependent manner, with noticeable protection at 25 μg/mL and almost complete prevention of DNA nicking at 200 μg/mL (Figure 3).

### 2.3. Immunolocalization Analysis of FgTGR

**Figure 4:**
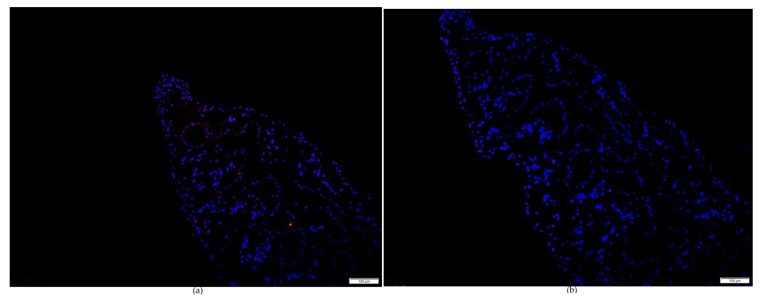
The localization analysis of FgTGR by immunofluorescence. (**a**) Probed with anti-FgTGR specific mouse serum. (**b**) Probed with the normal mouse serum.

Immunodetection for the TGR protein is shown in Figure 4. The sections were probed with the anti-FgTGR specific antibodies. In the section of adult parasites, a positive signal for FgTGR was detected. Distinct red fluorescence was observed throughout the entire adult specimen (Figure 4a). However, the negative control, using normal mouse serum, did not show a positive signal (Figure 4b).

### 2.4. Enzymatic Activities of FgTGRsec and FgTGR

**Figure 5:**
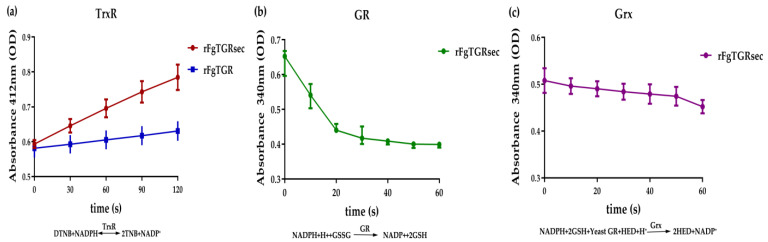
Absorbance of the activity detection analysis of FgTGR and FgTGRsec. (**a**) Absorbance of FgTGR and FgTGRsec at 412 nm for TrxR activity. (**b**) Absorbance of FgTGRsec at 340 nm for GR activity. (**c**) Absorbance of FgTGRsec at 340 nm for Grx activity.

For the activity of TrxR, an increased absorbance at 412 nm was observed (Figure 5a). The specific activities of FgTGR and FgTGRsec were 24.28 ± 1.51 U/mg and 54.47 ± 11.66 U/mg with DTNB, respectively. For the GR and Grx activities of FgTGRsec, a decrease at 340 nm was observed in GSSG and HED reduction, respectively (Figure 5b,c). The reaction formula for each enzyme activity is provided in Figure 5.

Table 1 summarizes the enzymatic activities and kinetic properties of recombinant TGR from two different parasites with various substrates. The kinetic parameters of the recombinant protein of FgTGRsec were also determined based on the method described by Kuntz et al. [12]. The TrxR and GR activities of FgTGRsec were higher than those of FhTGR. However, the Grx activity was lower than that of FhTGR. The kinetic parameter of FgTGRsec differed from that of FhTGR. The Km of FgTGRsec was 4-fold higher than FhTGR with DTNB as the substrate. The Km of FhTGR was lower than FgTGRsec with GSSG as the substrate. With HED as the substrate, FgTGRsec presented the lowest Km in the two different TGR.

### 2.5. Inhibtion of Catalytic Activity of FgTGRsec by Furoxan Derivatives

With furoxan as the positive control, the inhibition assay was performed to evaluate the effect of furoxan derivatives on the activity of FgTGRsec using different concentrations. As shown in Table 2, five furoxan derivatives showed a better inhibitory activity than furoxan. Notably, ZWJ-19 demonstrated a strong inhibitory effect on FgTGRsec, with an IC_50_ value of 3.82 μM, surpassing that of furoxan. Among the tested compounds, LGM-1 (IC_50_ = 6.39 μM) and LGM-35 (IC_50_ = 4.5 μM) also exhibited superior inhibitory activity compared with LGM-2 (IC_50_ = 15.19 μM) and CH-33 (IC_50_ = 18.54 μM). Apart from these five compounds, the remaining 57 furoxan derivatives did not show significant inhibition of the TrxR activity of FgTGRsec.

## 3. Discussion

### 3.1. Role of FgTGR in Antioxidant Defense and Redox Homeostasis

In tropical regions, *Fasciola* is a prevalent trematode parasite causing fascioliasis, which results in serious losses of domestic animals, especially cattle, sheep, and goats. The parasites can also infect humans. TCZ, an anthelmintic drug, is generally used to repeatedly treat fascioliasis. However, the treatment effect of TCZ is limited and temporary. Increasing incidences of TCZ resistance have been reported in several countries worldwide [4,27,28].

Reactive oxygen species (ROS) generally exist in normal metabolic living organisms. ROS accumulation can exert a toxic effect on biomolecules and subsequently cause serious oxidative damage to cells [29,30]. Reactive oxygen species can be generated in two ways: one occurs when the worms ingest erythrocytes, and the other arises from the adhesion of host effector cells to the worms. *F. gigantica* is a parasite that resides in the liver and bile ducts of its definitive host. While in the liver, the parasite is exposed to ROS produced by the host’s immune cells including neutrophils, eosinophils, and macrophages [31,32]. However, *F. gigantica* has developed antioxidant defenses and redox balance systems to protect itself from ROS [33,34]. Antioxidant proteins and enzymes in the parasites, including superoxide dismutase (SOD) [35], peroxiredoxin (Prx) [36,37], thioredoxin glutathione reductase (TGR) [38], thioredoxin (Trx) [16,39], and so on, could help them to escape the damage resulting from ROS. In *Fasciola*, TGR showed both Trx and GSH activities [10,40]. The Trx and GSH system consists of TrxR and GR, Grx, respectively [28]. TGR plays an essential role in the antioxidation system and is regarded as a potential target for drugs and a novel vaccine candidate against *F. gigantica*.

### 3.2. Structural and Functional Characteristics of FgTGR

In our previous study, FgTGRsec and FgTGR were purified using Ni-NTA affinity chromatography. The molecular weights of the two recombinant proteins were 72 kDa and 70 kDa, respectively [41]. However, FgTGR and FgTGRsec were homogenous, as indicated by single protein bands of ~66 kDa on SDS-PAGE in other works [16,28,42]. The results of these studies have been inconsistent due to variations in expression methods and plasmid systems. TGR contains two redox-active centers, specifically the -CXXXXC- and -GCUG- motifs, while Grx is characterized by a -CXXC- or -CXXS- motif as its redox-active site [43]. As shown in Figure 1, multiple sequence alignments highlight conserved sequences such as CPYC/CPHS, CVNVGC, and GCUG, marked with black boxes. However, Grx activity was absent in *Oesophagostomum dentatum* and *Tetrahymena thermophila* due to the lack of the -CXXC/S- motifs. As a result, their enzymes should be referred to as TrxR rather than TGR, despite being labeled as TGR in the NCBI database. In contrast, TGR in other parasites, such as *F. hepatica*, *S. japonicum*, *S. mansoni*, and *E. granulosus*, exhibits TrxR, GR, and Grx activities.

### 3.3. Comparative Enzymatic Activities of FgTGR and FgTGRsec

Several studies have highlighted TGR as a crucial enzyme for mitigating the toxicity of ROS in parasitic flukes [17,44,45,46]. To evaluate the DNA-protective activity of FgTGR, a DNA-nicking assay was performed using the MCO system. Reactive oxygen species generated by the MCO system caused damage to the plasmid pUC19. In this study, FgTGR demonstrated the ability to protect the plasmid from ROS-induced damage, with complete protection observed at a concentration of 200 µg/mL. In comparison, a concentration of 600 µg/mL was required for similar protection in *S. japonicum*.

All life stages of *F. gigantic* are exposed to ROS produced by the parasite’s metabolism, cell proliferation, and the host’s immune response. Immunohistochemistry revealed a broad distribution of FgTGR across all adult worm tissues, although its subunit structure was not identified. Changklungmoa et al. [16] also detected TGR expression in 2- and 4-week-old juveniles as well as adult worms of *F. gigantica*. The results further demonstrated that FgTGR is localized in the tegument and tegumental cells of juvenile worms. In adult parasites, FgTGR was detected in the tegument, parenchyma, eggs, testes, ovaries, and vitelline cells, but no positive signals were observed in the spines or muscles. The expression of FgTGR was notably higher in the reproductive organs, likely due to its involvement in reproduction and development. These findings suggest that FgTGR plays a critical role in protecting the parasites from ROS generated by the host’s immune cells.

TGR is a selenoprotein requiring a bacterial-type selenocysteine insertion sequence element (SECIS) and four gene products—selA, selB, selC, and selD—for the expression of recombinant FgTGRsec in prokaryotic systems [47,48]. In this study, the plasmids FgTGR-pET28a(+) and pSUABC were co-transformed into *E. coli* BL21 (DE3) cells, enabling the expression and purification of FgTGRsec. The recombinant FgTGRsec exhibited TrxR, GR, and Grx activities. Notably, FgTGRsec showed more than double the DTNB-based TrxR activity compared with FgTGR, which may be attributed to the presence of Sec and its higher affinity for DTNB. Among its activities, GR activity was the highest, whereas Grx activity was the lowest. Conversely, in *S. japonicum*, TGR displayed the highest Grx activity [11]. These findings underscore the role of Sec in enhancing TrxR and GR activities in FgTGRsec, consistent with prior studies [42,49]. Additionally, Kalita et al. [42] constructed a variant of FgTGRsec lacking the N-terminal Grx domain (CPYC) (∆NTD-FgTGRsec) to explore the domain’s function. The results showed that the Grx domain positively regulates TrxR activity in FgTGRsec. However, in certain platyhelminth parasites, the Grx domain does not influence TrxR activity but is crucial for GR activity [44]. This functional divergence may reflect the varying roles of the Grx domain across different platyhelminths.

TGR not only plays an important role in the redox biochemistry in several parasites [26,50,51], but is also essential for the survival of schistosomes. In *S. mansoni*, the parasites died within four days in vitro when the expression of TGR was silenced [12]. SjTGR may be an effective candidate antigen because of the decrease in the adult worm burden and the number of eggs [13]. The TrxR activity of TGR was inhibited by using siRNA to knock down the TGR gene in *S. japonicum* [17]. These studies demonstrated that TGR is a promising drug target for-parasitic diseases, hence, a number of inhibitors of TGR were screened. AF is a well-known inhibitor for TGR in many fluke parasites [11,12,19,52]. In *F. gigantica*, the inhibitory activity of FgTGRsec by AF was also reported, with an IC_50_ of 0.87 µM for TrxR activity and 8.3 µM for GR activity [42]. However, AF was also a potent inhibitor of TGR in *Taenia crassiceps cysticerci*. After culturing with AF for 12 h, the cysticerci were all dead, and the IC_50_ was only 3.8 μM [53]. In addition, oxadiazoles-2-oxides derivatives [14,54,55], isoxazolones, phosphinic acid amides, phosphoramidites [56], curcuminoids and curcumin derivatives [20], and so on [57], have been developed as inhibitors of TGR. In the study of oxadiazoles-2-oxides derivatives, it was found that furoxan could inhibit all stages of *S. mansoni* and kill the adult worms of *S. mansoni*, *S. japonicum*, and *Schistosoma haematobium* (*S. haematobium*), which are inhibitors of SmTGR [58]. In our previous study, we exploited a number of novel furoxan derivatives and evaluated the inhibitory activity against rSjTGRsec. Finally, a compound with trifuoromethyl on a pyridine ring showed an excellent inhibition of rSjTGRsec [14].

### 3.4. Inhibitory Effects of Furoxan Derivatives on FgTGRsec

In this study, a series of novel furoxan derivatives was evaluated for their ability to inhibit the DTNB-based TrxR activity of FgTGRsec. Among the 62 compounds tested, five—ZWJ-19, LGM-2, CH-33, LGM-1, and LGM-35—exhibited varying levels of inhibition. ZWJ-19 demonstrated the strongest inhibitory effect on TrxR activity, with an IC_50_ of 3.82 μM. In contrast, for *S. japonicum*, LGM-35 showed the most significant inhibition of TrxR activity [14], outperforming the furoxan derivatives previously tested in *S. mansoni* [58]. LGM-35 demonstrated an inhibitory effect against SjTGRsec; however, it was less effective than ZWJ-19 in *Fasciola gigantica*. This difference may be due to ZWJ-19’s chemical structure, which likely has a higher affinity for FgTGRsec compared with LGM-35. The amino acid sequence similarity between SjTGRsec and FgTGRsec was only 62.65%, leading to significant differences in protein folding, spatial structure and inhibitor binding sites. Consequently, the same furoxan derivative can exhibit varying levels of inhibition across different targets. At the highest tested concentration, the other 57 furoxan derivatives demonstrated less than 50% inhibition of FgTGRsec. The variability in inhibitory effects among compounds likely stems from differences in their chemical structures. Further exploration of the structure–activity relationship of these compounds is needed to better understand and optimize their inhibitory potential.

While this study focused specifically on the characterization of FgTGR and the identification of inhibitory furoxan derivatives for *F. gigantica*, the implications of these findings may extend to the closely related and economically significant parasite, *Fasciola hepatica*. The exceptionally high sequence identity (97.99%) and similar kinetic profiles between FgTGR and FhTGR [10,41] provide a strong rationale for the potential cross-reactivity of the identified inhibitors. The demonstrated efficacy of other TGR inhibitors, such as auranofin, across multiple trematode species further supports this concept [10,12,17]. A limitation of this study is that the inhibitory efficacy of the furoxan derivatives was not experimentally validated against FhTGR or in *F. hepatica* in vitro/in vivo models. Therefore, future work will be essential to directly evaluate the potency of lead compounds like ZWJ-19 against *F. hepatica*, which would significantly broaden the impact of this study for veterinary medicine and animal welfare.

## 4. Materials and Methods

### 4.1. Parasites, Proteins, and Antibodies

The adult worms of *F. gigantica* were collected from the infected liver of buffaloes in a slaughterhouse in Anhui Province. The worms were washed thoroughly with cold phosphate-buffered saline (PBS, pH 7.4) and stored. The animals were infected naturally and part of the normal work in the slaughterhouse. The recombinant FgTGR and FgTGRsec were successfully expressed and purified in our previous study [41]. The polyclonal antibodies of mice against FgTGR were prepared and stored at the Shanghai Veterinary Research Institute, Chinese Academy of Agricultural Sciences.

### 4.2. Sequence and Phylogenetic Analyses of FgTGR

The nucleotide-deduced amino acid sequences and their identities were analyzed by using BLAST in NCBI (The National Center for Biotechnology Information, NCBI, https://blast.ncbi.nlm.nih.gov/Blast.cgi?PROGRAM=blastp&PAGE_TYPE=BlastSearchLINK_LLOC=blasthome=blasthome, accessed on 2 June 2025) and DNAstar 7.0 software, respectively. Multiple sequence alignment for amino acid sequences of the TGR from different parasites and host species was generated with ClustalW. The MEGA 11 program (neighbor-joining method) was used to analyze the phylogeny of the different TGR amino acid sequences. The signal peptide of these sequences was predicted by the SignalP 3.0 server (https://services.healthtech.dtu.dk/services/SignalP-3.0/, accessed on 2 June 2025).

### 4.3. Localization of FgTGR Protein by Immunolocalization

Paraffin sections (5 μm) of *F. gigantica* adult worms were treated with xylene twice for 10 min each. The sections were rehydrated in ethanol 100%, 95%, 80%, and 70% for 5 min each, and then treated with ddH_2_O twice for 5 min each. The sections were microwaved at 100 °C in trisamine-ethylene diamine tetra-acetic acid (Tris-EDTA) for 20 min for antigen renaturation. The tissue sections were blocked with 3% BSA for 30 min at room temperature to prevent non-specific binding, followed by overnight incubation at 4 °C with the primary antibody (mouse serum specific to rFgTGR, diluted at 1:200). Subsequently, the sections were incubated with goat anti-mouse IgG (1:1000, Invitrogen, Waltham, MA, USA) for 1 h at room temperature. Finally, the sections were stained with 4’,6 -diamidino-2-phenylindole (DAPI) for 10 min in the dark. PBST was used to wash the sections three times for 5 min between every step. The samples were detected and analyzed for the distribution of TGR using a fluorescent microscope (Nikon, Japan). The non-immunized serum was used as a negative control, and the process of the negative experiment was the same as the above description.

### 4.4. Antioxidant Activity of FgTGR

A metal-catalyzed oxidation (MCO) DNA-cleavage (nicking) assay was performed as previously described [59,60], with some modifications. First, a reaction mixture (50 μL) containing different concentrations of FgTGR (12.5–200 μg/mL), 33 μM FeCl_3_, and 3.3 mM dithiothreitol (DTT) was incubated at 37 °C. After 2 h, the pUC19 supercoiled plasmid DNA (300 ng) was added to the mixtures, and then the mixtures were incubated at 37 °C for 2.5 h. Subsequently, the results were detected by agarose gel electrophoresis. The concentration of purified protein FgTGR was 600 μg/mL, and bovine serum albumin (BSA) (600 μg/mL) was used as a control protein. The process of the control experiment was the same as the above description.

### 4.5. Enzymatic Assays

#### 4.5.1. TrxR Activity Assay of FgTGRsec

The TrxR activity of FgTGR and FgTGRsec was determined by using the 5, 5′-diothiobis (2-nitrobenzoic acid, DTNB, 4 mg/mL) (Sigma, St. Louis, MO, USA) reduction assay at room temperature. The reaction mixture was composed of 180 μL working buffer (0.1 mM potassium phosphate, pH 7.0, 10 mMEDTA, 100 μM β-nicotinamide adenine dinucleotide phosphate (NADPH) (Sigma)), 4 μL assay buffer (0.1 mM potassium phosphate, pH 7.0, 10 mM EDTA), 6 μL DTNB, and 10 μL rFgTGR or rFgTGRsec (600 μg/mL). The reaction was initiated by the adding DTNB at concentrations ranging from 100 μmol/L to 1000 μmol/L. An increase in absorbance at 412 nm was observed within the first 2 min. One unit of thioredoxin reductase (TrxR) activity was defined as the amount of enzyme that catalyzes the production of 1 μmol of 2-nitro-5-thiobenoic acid (TNB) per minute at room temperature. The reaction mixture without FgTGR or FgTGRsec was used as a negative control, and the processes of the negative experiments were the same as the above description.

#### 4.5.2. GR Assay of FgTGRsec

The activity of GR was determined using oxidized glutathione (GSSG). The reaction mixture contained 10 µL FgTGRsec, 200 µM NADPH, and 1 mM EDTA in 20 mM potassium phosphate (pH 7.0). The reaction was initiated by the addition of NADPH. The GSSG concentration was varied from 0.5 µM to 5 µM. The consumption of NADPH was monitored by observing the decrease during the first 1 min in A340 (ε340 nm = 6.22 mM^−1^ cm^−1^). One unit of GR activity was defined as the oxidation of 1 µmol of NADPH per min at room temperature.

#### 4.5.3. Grx Assay of FgTGRsec

Glutaredoxin (Grx) activity was assayed using β-hydroxyethyl disulfide (HED) as the substrate. The Grx activity reaction mixture contained 1 mM EDTA in 20 mM potassium phosphate (pH 7.0), 100 µM NADPH, 1 mM glutathione (GSH), 8 mM HED, and 0.6 units of yeast GR. The reaction was initiated by the addition of the recombinant protein FgTGRsec. The GSH and HED concentrations were varied from 100 µM to 1500 µM and 1 mM to 8 mM, respectively. The activity of Grx was determined due to the consumption of NADPH by monitoring the decrease at 340 nm during the first 1 min (ε340 nm = 6.22 mM^−1^ cm^−1^). One unit of Grx activity was defined as the oxidation of 1 µmol of NADPH per min at room temperature.

### 4.6. Compound Inhibition of FgTGRsec

To evaluate the effect of furoxan derivatives on FgTGRsec, the activity of TrxR was measured. The furoxan derivatives were soluble in dimethyl sulfoxide (DMSO), and seven concentrations of each derivative were prepared based on their distinct structures. For the DTNB-based TrxR activity inhibition assay, reaction mixtures were prepared as described in the TrxR activity assay protocol. The components were incubated for 20 min, after which the reaction was initiated by adding DTNB. The increase in absorbance at 412 nm was measured to evaluate the inhibitory effect of each furoxan derivative. The half-maximal inhibitory concentration (IC_50_) values of the compounds were calculated by GraphPad Prism v6.0c software. The reaction without FgTGRsec and furoxan derivatives served as the blank control, while the reaction with furoxan was the positive control. All assays were performed in triplicate.

### 4.7. Statistical Analysis

Data were expressed as the mean ± standard deviation and analyzed using GraphPad Prism version 8.0c (GraphPad Software, San Diego, CA, USA) from independent experiments. The normality of data distribution was evaluated using appropriate tests prior to statistical comparisons. For datasets demonstrating normal distribution, Duncan’s multiple range *t*-test was employed to compare differences among the experimental groups including enzymatic activity assays and inhibition assays of FgTGRsec by furoxan derivatives. In contrast, for data that deviated from the normal distribution, the non-parametric Kruskal–Wallis test was applied. A *p*-value of less than 0.05 (*p* < 0.05) was considered statistically significant.

## 5. Conclusions

TGR serves as a central enzyme linking the Trx and GSH systems in many parasites. Numerous studies have highlighted its significance as a potential target for developing new drugs against fascioliasis. In this study, we characterized the biochemical properties of two distinct variants of TGR in *Fasciola gigantica*. FgTGRsec was shown to play a crucial role in maintaining redox balance as an antioxidant enzyme. By assessing its activity with various substrates, we demonstrated that the presence of Sec was essential for the enhanced TrxR activity of FgTGRsec. Additionally, we identified five furoxan derivatives capable of inhibiting FgTGRsec TrxR activity, with ZWJ-19 exhibiting the strongest inhibition. These findings suggest that ZWJ-19 holds promise as a lead compound for further investigation in *F. gigantica*. Given the unique properties of TGR, it represents a promising target for the development of future therapies against fascioliasis.

## Figures and Tables

**Table 1:** Comparison of enzyme activity and kinetic properties of recombinant TGR from *F. gigantica* [26] and *F. hepatica* with different substrates.

Property	Substrate	*F. gigantica* TGRsec	*F. hepatica* TGR
Specific actives (U/mg)	DTNB	54.47 ± 11.66	10.2 ± 1.4
	GSSG	71.14 ± 11.48	64.5 ± 5.5
	HED	11.86 ± 1.88	55.4 ± 6
Apparent Km (µM)	DTNB	189.39 ± 52.90	46 ± 12
	GSSG	71.14 ± 11.48	30 ± 5
	HED	11.86 ± 78.93	—

**Table 2:** Structures of the furoxan derivatives and their inhibitory activity against the TrxR of FgTGResc.

Compounds	Structure	IC_50_ of rFgTGRsec (μM)
LGM-1	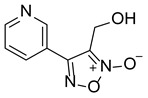	6.93 ± 1.74
LGM-2	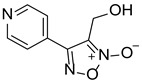	15.19 ± 0.80
LGM-35	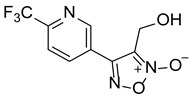	4.5 ± 0.35
ZWJ-19	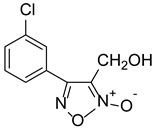	3.82 ± 0.33
CH-33	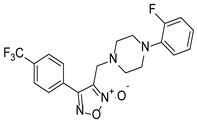	18.54 ± 2.57

## Data Availability

All data related to the research are included in the article.

## References

[1] Renslo A.R., McKerrow J.H. (2006). Drug discovery and development for neglected parasitic diseases. Nat. Chem. Biol..

[2] Robinson M.W., Dalton J.P. (2009). Zoonotic helminth infections with particular emphasis on fasciolosis and other trematodiases. Philos. Trans. R. Soc. Lond. B Biol. Sci..

[3] Ferraro F., Corvo I., Bergalli L., Ilarraz A., Cabrera M., Gil J., Susuki B.M., Caffrey C.R., Timson D.J., Robert X. (2020). Novel and selective inactivators of Triosephosphate isomerase with anti-trematode activity. Sci. Rep..

[4] Kelley J.M., Elliott T.P., Beddoe T., Anderson G., Skuce P., Spithill T.W. (2016). Current Threat of Triclabendazole Resistance in *Fasciola hepatica*. Trends Parasitol..

[5] Thakare R., Dasgupta A., Chopra S. (2019). Triclabendazole for the treatment of fascioliasis. Drugs Today.

[6] Olaechea F., Lovera V., Larroza M., Raffo F., Cabrera R. (2011). Resistance of *Fasciola hepatica* against triclabendazole in cattle in Patagonia (Argentina). Vet. Parasitol..

[7] Cabada M.M., Lopez M., Cruz M., Delgado J.R., Hill V., White A.C. (2016). Treatment Failure after Multiple Courses of Triclabendazole among Patients with Fascioliasis in Cusco, Peru: A Case Series. PLoS Negl. Trop. Dis..

[8] Tripathi T., Suttiprapa S., Sripa B. (2017). Unusual thiol-based redox metabolism of parasitic flukes. Parasitol. Int..

[9] Bonilla M., Denicola A., Marino S.M., Gladyshev V.N., Salinas G. (2011). Linked thioredoxin-glutathione systems in platyhelminth parasites: Alternative pathways for glutathione reduction and deglutathionylation. J. Biol. Chem..

[10] Salinas G., Selkirk M.E., Chalar C., Maizels R.M., Fernandez C. (2004). Linked thioredoxin-glutathione systems in platyhelminths. Trends Parasitol..

[11] Song L., Li J., Xie S., Qian C., Wang J., Zhang W., Yin X., Hua Z., Yu C. (2012). Thioredoxin glutathione reductase as a novel drug target: Evidence from *Schistosoma japonicum*. PLoS ONE.

[12] Kuntz A.N., Davioud-Charvet E., Sayed A.A., Califf L.L., Dessolin J., Arner E.S., Williams D.L. (2007). Thioredoxin glutathione reductase from *Schistosoma mansoni*: An essential parasite enzyme and a key drug target. PLoS Med..

[13] Han Y., Zhang M., Hong Y., Zhu Z., Li D., Li X., Fu Z., Lin J. (2012). Characterization of thioredoxin glutathione reductase in *Schiotosoma japonicum*. Parasitol. Int..

[14] Li G., Guo Q., Feng C., Chen H., Zhao W., Li S., Hong Y., Sun D. (2021). Synthesis of oxadiazole-2-oxide derivatives as potential drug candidates for schistosomiasis targeting SjTGR. Parasit. Vectors.

[15] Tang W., Yue Y., Shi B., Zhao X., Hong Y. (2025). Characterization and enzymatic function of thioredoxin glutathione reductase in Orientobilharzia turkestanicum isolated from Xizang. BMC Vet. Res..

[16] Changklungmoa N., Kueakhai P., Sangpairoj K., Chaichanasak P., Jaikua W., Riengrojpitak S., Sobhon P., Chaithirayanon K. (2015). Molecular cloning and characterization of *Fasciola gigantica* thioredoxin-glutathione reductase. Parasitol. Res..

[17] Han Y., Fu Z., Hong Y., Zhang M., Han H., Lu K., Yang J., Li X., Lin J. (2014). Inhibitory effects and analysis of RNA interference on thioredoxin glutathione reductase expression in *Schistosoma japonicum*. J. Parasitol..

[18] Prum S., Plumworasawat S., Chaiyadet S., Saichua P., Thanan R., Laha T., Laohaviroj M., Sripa B., Suttiprapa S. (2020). Characterization and in vitro functional analysis of thioredoxin glutathione reductase from the liver fluke *Opisthorchis viverrini*. Acta Trop..

[19] Caroli A., Simeoni S., Lepore R., Tramontano A., Via A. (2012). Investigation of a potential mechanism for the inhibition of SmTGR by Auranofin and its implications for *Plasmodium falciparum* inhibition. Biochem. Biophys. Res. Commun..

[20] Guevara-Flores A., Martinez-Gonzalez J.J., Herrera-Juarez A.M., Rendon J.L., Gonzalez-Andrade M., Torres Duran P.V., Enriquez-Habib R.G., Del Arenal Mena I.P. (2019). Effect of curcuminoids and curcumin derivate products on thioredoxin-glutathione reductase from *Taenia crassiceps cysticerci*. Evidence suggesting a curcumin oxidation product as a suitable inhibitor. PLoS ONE.

[21] Shukla R., Shukla H., Kalita P., Tripathi T. (2018). Structural insights into natural compounds as inhibitors of *Fasciola gigantica* thioredoxin glutathione reductase. J. Cell Biochem..

[22] Neves B.J., Dantas R.F., Senger M.R., Melo-Filho C.C., Valente W.C., de Almeida A.C., Rezende-Neto J.M., Lima E.F., Paveley R., Furnham N. (2016). Discovery of New Anti-Schistosomal Hits by Integration of QSAR-Based Virtual Screening and High Content Screening. J. Med. Chem..

[23] Li T., Ziniel P.D., He P.Q., Kommer V.P., Crowther G.J., He M., Liu Q., Van Voorhis W.C., Williams D.L., Wang M.W. (2015). High-throughput screening against thioredoxin glutathione reductase identifies novel inhibitors with potential therapeutic value for schistosomiasis. Infect. Dis. Poverty.

[24] Lyu H., Petukhov P.A., Banta P.R., Jadhav A., Lea W.A., Cheng Q., Arner E.S.J., Simeonov A., Thatcher G.R.J., Angelucci F. (2020). Characterization of Lead Compounds Targeting the Selenoprotein Thioredoxin Glutathione Reductase for Treatment of Schistosomiasis. ACS Infect. Dis..

[25] Ross F., Hernandez P., Porcal W., Lopez G.V., Cerecetto H., Gonzalez M., Basika T., Carmona C., Flo M., Maggioli G. (2012). Identification of thioredoxin glutathione reductase inhibitors that kill cestode and trematode parasites. PLoS ONE.

[26] Guevara-Flores A., Pardo J.P., Rendon J.L. (2011). Hysteresis in thioredoxin-glutathione reductase (TGR) from the adult stage of the liver fluke *Fasciola hepatica*. Parasitol. Int..

[27] Brennan G.P., Fairweather I., Trudgett A., Hoey E., Coy M., McConville M., Meaney M., Robinson M., McFerran N., Ryan L. (2007). Understanding triclabendazole resistance. Exp. Mol. Pathol..

[28] Gupta A., Kesherwani M., Velmurugan D., Tripathi T. (2016). *Fasciola gigantica* thioredoxin glutathione reductase: Biochemical properties and structural modeling. Int. J. Biol. Macromol..

[29] Yu B.P. (1994). Cellular defenses against damage from reactive oxygen species. Physiol. Rev..

[30] Nikapitiya C., De Zoysa M., Whang I., Kim C.G., Lee Y.H., Kim S.J., Lee J. (2009). Molecular cloning, characterization and expression analysis of peroxiredoxin 6 from disk abalone Haliotis discus discus and the antioxidant activity of its recombinant protein. Fish Shellfish Immunol..

[31] Bertini R., Howard O.M., Dong H.F., Oppenheim J.J., Bizzarri C., Sergi R., Caselli G., Pagliei S., Romines B., Wilshire J.A. (1999). Thioredoxin, a redox enzyme released in infection and inflammation, is a unique chemoattractant for neutrophils, monocytes, and T cells. J. Exp. Med..

[32] Nakamura H., Herzenberg L.A., Bai J., Araya S., Kondo N., Nishinaka Y., Yodoi J. (2001). Circulating thioredoxin suppresses lipopolysaccharide-induced neutrophil chemotaxis. Proc. Natl. Acad. Sci. USA.

[33] Wang Y., Feinstein S.I., Manevich Y., Ho Y.S., Fisher A.B. (2006). Peroxiredoxin 6 gene-targeted mice show increased lung injury with paraquat-induced oxidative stress. Antioxid. Redox Signal.

[34] Suttiprapa S., Loukas A., Laha T., Wongkham S., Kaewkes S., Gaze S., Brindley P.J., Sripa B. (2008). Characterization of the antioxidant enzyme, thioredoxin peroxidase, from the carcinogenic human liver fluke, *Opisthorchis viverrini*. Mol. Biochem. Parasitol..

[35] Assady M., Farahnak A., Golestani A., Esharghian M. (2011). Superoxide Dismutase (SOD) Enzyme Activity Assay in *Fasciola spp*. Parasites and Liver Tissue Extract. Iran. J. Parasitol..

[36] Sangpairoj K., Changklungmoa N., Vanichviriyakit R., Sobhon P., Chaithirayanon K. (2014). Analysis of the expression and antioxidant activity of 2-Cys peroxiredoxin protein in *Fasciola gigantica*. Exp. Parasitol..

[37] Chaithirayanon K., Sobhon P. (2010). Molecular cloning and characterization of two genes encoding 2-Cys peroxiredoxins from *Fasciola gigantica*. Exp. Parasitol..

[38] Maggioli G., Silveira F., Martin-Alonso J.M., Salinas G., Carmona C., Parra F. (2011). A recombinant thioredoxin-glutathione reductase from *Fasciola hepatica* induces a protective response in rabbits. Exp. Parasitol..

[39] Alger H.M., Sayed A.A., Stadecker M.J., Williams D.L. (2002). Molecular and enzymatic characterisation of *Schistosoma mansoni* thioredoxin. Int. J. Parasitol..

[40] Angelucci F., Miele A.E., Boumis G., Dimastrogiovanni D., Brunori M., Bellelli A. (2008). Glutathione reductase and thioredoxin reductase at the crossroad: The structure of *Schistosoma mansoni* thioredoxin glutathione reductase. Proteins.

[41] He Y., Guo Q., Yong C., Jia J., Han Y., Zhou Y., Lu L., Tang W., Hong Y., Fu Z. (2024). Expression, Purification and Preparation of Polyclonal Antibodies of Thioredoxin Glutathione Reductase from *Fasciola gigantica*. Chin. J. Zoonotic Infect. Dis..

[42] Kalita P., Shukla H., Shukla R., Tripathi T. (2018). Biochemical and thermodynamic comparison of the selenocysteine containing and non-containing thioredoxin glutathione reductase of *Fasciola gigantica*. Biochim. Biophys. Acta Gen. Subj..

[43] Angelucci F., Dimastrogiovanni D., Boumis G., Brunori M., Miele A.E., Saccoccia F., Bellelli A. (2010). Mapping the catalytic cycle of Schistosoma mansoni thioredoxin glutathione reductase by X-ray crystallography. J. Biol. Chem..

[44] Williams D.L., Bonilla M., Gladyshev V.N., Salinas G. (2013). Thioredoxin glutathione reductase-dependent redox networks in platyhelminth parasites. Antioxid. Redox Signal.

[45] Mkoji G.M., Smith J.M., Prichard R.K. (1988). Antioxidant systems in *Schistosoma mansoni* correlation between susceptibility to oxidant killing and the levels of scavengers of hydrogen peroxide and oxygen free radicals. Int. J. Parasitol..

[46] Hong Y., Han Y., Fu Z., Han H., Qiu C., Zhang M., Yang J., Shi Y., Li X., Lin J. (2013). Characterization and expression of the Schistosoma japonicum thioredoxin peroxidase-2 gene. J. Parasitol..

[47] Arner E.S., Sarioglu H., Lottspeich F., Holmgren A., Bock A. (1999). High-level expression in Escherichia coli of selenocysteine-containing rat thioredoxin reductase utilizing gene fusions with engineered bacterial-type SECIS elements and co-expression with the selA, selB and selC genes. J. Mol. Biol..

[48] Rengby O., Johansson L., Carlson L.A., Serini E., Vlamis-Gardikas A., Karsnas P., Arner E.S. (2004). Assessment of production conditions for efficient use of *Escherichia coli* in high-yield heterologous recombinant selenoprotein synthesis. Appl. Env. Microbiol..

[49] Zhong L., Arner E.S., Ljung J., Aslund F., Holmgren A. (1998). Rat and calf thioredoxin reductase are homologous to glutathione reductase with a carboxyl-terminal elongation containing a conserved catalytically active penultimate selenocysteine residue. J. Biol. Chem..

[50] Agorio A., Chalar C., Cardozo S., Salinas G. (2003). Alternative mRNAs arising from trans-splicing code for mitochondrial and cytosolic variants of *Echinococcus granulosus* thioredoxin *Glutathione reductase*. J. Biol. Chem..

[51] Guevara-Flores A., Del Arenal I.P., Mendoza-Hernandez G., Pardo J.P., Flores-Herrera O., Rendon J.L. (2010). Mitochondrial Thioredoxin-Glutathione Reductase from Larval *Taenia crassiceps* (Cysticerci). J Parasitol Res..

[52] Angelucci F., Sayed A.A., Williams D.L., Boumis G., Brunori M., Dimastrogiovanni D., Miele A.E., Pauly F., Bellelli A. (2009). Inhibition of *Schistosoma mansoni* thioredoxin-glutathione reductase by auranofin: Structural and kinetic aspects. J. Biol. Chem..

[53] Martinez-Gonzalez J.J., Guevara-Flores A., Alvarez G., Rendon-Gomez J.L., Del Arenal I.P. (2010). In vitro killing action of auranofin on *Taenia crassiceps* metacestode (cysticerci) and inactivation of thioredoxin-glutathione reductase (TGR). Parasitol. Res..

[54] Rai G., Sayed A.A., Lea W.A., Luecke H.F., Chakrapani H., Prast-Nielsen S., Jadhav A., Leister W., Shen M., Inglese J. (2009). Structure mechanism insights and the role of nitric oxide donation guide the development of oxadiazole-2-oxides as therapeutic agents against schistosomiasis. J. Med. Chem..

[55] Song L.J., Luo H., Fan W.H., Wang G.P., Yin X.R., Shen S., Wang J., Jin Y., Zhang W., Gao H. (2016). Oxadiazole-2-oxides may have other functional targets, in addition to SjTGR, through which they cause mortality in *Schistosoma japonicum*. Parasit. Vectors.

[56] Huang J., Hua W., Li J., Hua Z. (2015). Molecular docking to explore the possible binding mode of potential inhibitors of thioredoxin glutathione reductase. Mol. Med. Rep..

[57] Eweas A.F., Allam G. (2018). Targeting thioredoxin glutathione reductase as a potential antischistosomal drug target. Mol. Biochem. Parasitol..

[58] Sayed A.A., Simeonov A., Thomas C.J., Inglese J., Austin C.P., Williams D.L. (2008). Identification of oxadiazoles as new drug leads for the control of schistosomiasis. Nat. Med..

[59] Lim Y.S., Cha M.K., Kim H.K., Uhm T.B., Park J.W., Kim K., Kim I.H. (1993). Removals of hydrogen peroxide and hydroxyl radical by thiol-specific antioxidant protein as a possible role In Vivo. Biochem. Biophys. Res. Commun..

[60] Sauri H., Butterfield L., Kim A., Shau H. (1995). Antioxidant function of recombinant human natural killer enhancing factor. Biochem. Biophys. Res. Commun..

